# Process Evaluation of the Implementation of the Secondary 2 Program of Project P.A.T.H.S. in the Experimental Implementation Phase

**DOI:** 10.1100/tsw.2008.23

**Published:** 2008-01-14

**Authors:** Daniel T. L. Shek, Tak Yan Lee, Rachel C. F. Sun

**Affiliations:** ^1^ Centre for Quality of Life, Hong Kong Institute of Asia-Pacific Studies, The Chinese University of Hong Kong, Hong Kong; ^2^ Kiang Wu Nursing College of Macau, Macau; ^3^ Social Welfare Practice and Research Centre, The Chinese University of Hong Kong, Hong Kong; ^4^ Department of Applied Social Studies, City University of Hong Kong, Hong Kong

**Keywords:** process evaluation, adolescents, positive youth development program

## Abstract

This study aimed to understand the implementation quality of the Tier 1 Program (Secondary 2 Curriculum) delivered in the Experimental Implementation Phase of the Project P.A.T.H.S. (Positive Adolescent Training through Holistic Social Programmes). Observers carried out process evaluation in the form of systematic observations of curriculum units in four randomly selected schools. Results showed that the overall level of program adherence was generally high, ranging from 70 to 95%, with an average of 83.6%. The mean ratings of the program implementation quality were high, and the inter-rater reliability on these ratings across the observers was highly reliable. Despite limitations, the findings of this study suggest that the implementation quality of the Secondary 2 Program (Tier 1 Program) of the Experimental Implementation Phase was favorable, and provide supporting evidence to account for the successful and encouraging outcomes of a major positive youth development program in Hong Kong.

## INTRODUCTION

In the context of evaluation of positive youth development programs, a survey of the literature shows that findings on program implementation quality are rarely reported[1,2,3]. Because most evaluation studies focused primarily on objective and subjective outcome evaluation, recent process evaluation studies represent a concerted effort to fill in the gap[4,5].

Shek et al.[4] pointed out several fatal consequences of overlooking the quality of the implementation of a program. First, the "black box" approach would make it difficult to understand the process of the program success or failure. Second, the lack of process evaluation would prevent the program developers from looking at the strengths and weaknesses of the programs developed. Third, the developers and implementers could not effectively decide how the program would be more effective if implemented again.

Finally, without process evaluation, program developers would have to wait until the outcome data are collected if they wished to refine the program.

Against the above background, there are several arguments for conducting process evaluation[6]. First, process evaluation can tell the program developers whether a Type III error (i.e., existence or nonexistence of program effect because of occurrence of activities different from those intended by the program developers) has occurred. Second, fidelity in program implementation can be promoted by feedback collected in the implementation process. Third, process evaluation can help program developers to understand whether the intended targets receive the program. Fourth, it can help to identify factors that contribute to program success or failure. Finally, program developers can use process evaluation findings to understand how the developed program can be implemented successfully in human organizations and communities, which are always complex in nature. Weinbach[7] provided two further reasons to support conducting a process evaluation. First, it can provide some valuable insights about a program that might be overlooked by the program developers. Second, it examines a program somewhat broadly, like a "systems analysis", to examine how the program works overall.

In its broad sense, the central research question of a process evaluation is: What happened and why did it happen? The implicit research hypothesis is: Something happened that affected the program's ability to achieve its outcomes. Other specific research questions for a process evaluation can also be used[7, p.168], including: How did the program come into existence in the first place? What changes occurred over time that were unplanned and inconsistent with the program model? What should be done differently if a similar program is to be undertaken? The present study focuses on a central research question, i.e., Are program activities being carried out as intended? The research hypothesis is that program activities are being accomplished as intended. Besides, the question regarding the quality of implementation was asked.

In Hong Kong, primary prevention programs targeting specific adolescent developmental problems and positive youth development programs are called for[8] in view of the worrying trends and phenomena related to the development of adolescents, such as mental health problems, abuse of psychotropic substances, adolescent suicide, school violence, and drop in family solidarity. However, a review of the literature shows that there are very few systematic and multiyear positive youth development programs in Hong Kong. Even if such programs exist, they commonly deal with isolated problems and issues in adolescent development (i.e., deficits-oriented programs) and they are relatively short term in nature[4]. In addition, systematic and long-term evaluation of the available programs does not exist.

To promote holistic development among adolescents in Hong Kong, the Hong Kong Jockey Club Charities Trust approved HK$400 million to launch a project entitled "P.A.T.H.S. to Adulthood: A Jockey Club Youth Enhancement Scheme". "P.A.T.H.S." denotes Positive Adolescent Training through Holistic Social Programmes. The Trust invited academics of five universities in Hong Kong to form a research team, with The Chinese University of Hong Kong as the lead institution, to develop a multiyear universal positive youth development program (Tier 1 Program) to promote holistic adolescent development in Hong Kong, to provide training for teachers and social workers who implement the program, and to carry out longitudinal evaluation of the project.

There are two implementation phases in this project – Experimental Implementation Phase (EIP) and Full Implementation Phase (FIP). For the EIP (January 2006 to August 2008), 52 secondary schools were invited to participate in the project with the objectives of accumulating experience in program implementation, and familiarizing front-line workers with the program design and philosophy. In the 2006/07 school year, the programs were implemented on a full scale at the Secondary 1 level (FIP-S1: 2006–07). In the 2007/08 school year, the programs are being implemented at the Secondary 1 and 2 levels. In the 2008/09 school year, the programs will be implemented at the Secondary 1, 2, and 3 levels.

The Tier 1 Program is a universal positive youth development program where students in Secondary 1 to 3 will participate in the program, normally with 20 h of training in the school year at each grade. The program was constructed based on the 15 positive youth development constructs described in Catalano et al.[9]. A summary of the constructs, aims, and learning targets of the curriculum units for Secondary 2 is presented in Table 1. Apart from the positive youth development constructs, the ecological perspective

**TABLE 1**
**An Overview of the 20-h Full Program of Secondary 2 Curriculum**

| Construct | Unit | Unit Aims | Learning Targets |
|---|---|---|---|
| 1. Bonding | What Can I Do for My Family? (BO2.1) (30 min) | To encourage students to make more contributions at home so as to strengthen their relationship with the family | To encourage students to review the occasions in which they received care from their parents; to explore the roles students may play at home and to enhance their motivation to contribute to their families |
| | Parents' Messages (BO2.2) (30 min) | To learn the skills that lead to better communication with parents and to strengthen parent-child relationships | To analyze parents' messages from person-oriented and task-oriented points of view; to learn to respond to parents' messages properly and establish a good relationship with parents |
| 2. Social competence | Who Is a Hong Konger? (SC2.1) (30 min) | To know why discrimination against people of different races is not desirable; to know more about ethnic minorities in Hong Kong; to learn to accept, value, and respect different ethnic groups | To know about the different ethnic groups in Hong Kong; to learn to accept people coming from different ethnic groups and to appreciate and value their qualities |
| | A Career Rhapsody (SC2.2) (30 min) | To build a vision among students that we can contribute to Hong Kong by fulfilling one's duty; to build up the value that people of different occupations should be accorded the same respect | To understand that different occupations contribute to Hong Kong and should be respected; to build up the value that we should respect people from all walks of life, we can make contribution to Hong Kong by fulfilling our duty |
| 3. Emotional competence | My Soul Mate (EC2.1) (30 min) | To increase students' abilities in understanding others' emotions and feelings | To be empathetic towards others' feelings; to understand the importance of respecting others' emotions |
| | Reasonable Response (EC2.2) (30 min) | To help students understand how negative emotions affect their relationships | To understand how improper negative emotional expressions affect interpersonal relationships; to recognize the importance of properly expressing negative emotions |
| | Two are Better than One (EC2.3) (30 min) | To encourage students to share distress with parents and family members | To identify the characteristics of parent-child communication; to list appropriate skills for sharing with parents |
| | A Perfect Match (EC2.4) (30 min) | To encourage students to share distress with friends | To identify characteristics of friends for secure sharing and factors to consider in sharing; to develop basic active-listening and response skills |
| 4. Cognitive competence | Thinking Styles Unveiled (CC2.1) (30 min) | To cultivate creative thinking skills through exposure to different types of thinking styles | To identify the characteristics of different types of thinking styles; to identify different thinking styles pertaining to various occupations |
| | Creator of the New Century (CC2.2) (30 min) | To cultivate creative thinking skills | To apply different thinking styles in creative problem solving |
| 5. Behavioral competence | Friendly Criticism? (BC2.1) (30 min) | To understand that criticism may be given with good and/or bad intentions; to learn how to criticize out of goodwill | To understand that criticism may be given with good and/or bad intentions; to understand that the method of giving criticism may affect the receptivity of the criticized; to learn how to criticize out of goodwill and avoid misunderstanding |

**TABLE 1 (continued)**

| Construct | Unit | Unit Aims | Learning Targets |
|---|---|---|---|
| | I Want an Apology! (BC2.2) (30 min) | To understand that appropriately handling mistakes (both ours and others') may avoid harming interpersonal relationships | To learn to face and handle improper behavior against us; to learn how to apologize to others sincerely |
| 6. Moral competence | In the Restaurant (MC2.1) (30 min) | To understand that under all circumstances, with or without laws, maintaining fairness requires us to consider the whole situation and the needs of all the parties involved | To learn to respect others and be considerate about their needs and feelings when seeking fairness; to understand that seeking fairness does not mean that everyone gets what he/she wants; to understand that seeking fairness when no laws apply does not mean fighting for what we want |
| | Seat Fighter (MC2.2) (30 min) | To increase students' awareness of helping others and help them to understand that goodwill is what matters when offering help | To learn to consider the needs of others and take the initiative to help others by examining selfish behaviors; to learn how to help others bravely, wisely, and skillfully |
| 7. Self-efficacy | Learning Tactics (SE2.1) (30 min) | To enhance students' academic self-efficacy | To explore different learning tactics; to discover suitable learning tactics |
| | The Warped Monster House (SE2.2) (30 min) | To help students lessen the negative impact of distorted thoughts on their self-efficacy, in order to prevent depression | To identify distorted thoughts; to learn to penetrate and deal with distorted thoughts |
| 8. Prosocial norms | Different Identities, Different Duties (PN2.1) (30 min) | To understand that everyone has his/her own social responsibility | To understand that social norms may or may not apply to people with different identities when faced with the same social issue or situation; to understand that people of different age groups and/or backgrounds have different social responsibilities |
| | You Better Do What People Tell You? (PN2.2) (30 min) | To identify different societal expectations toward conforming behavior | To realize that society may have different expectations of prosocial behavior for people of different age groups and/or backgrounds; to realize that conforming behavior could be good or bad; to understand that individuals should make decisions rationally rather than conforming without thought |
| | Fit for What? (PN2.3) (30 min) | To identify the incentives for complying with social norms and the disincentives of violating those norms | To develop the ability to identify incentives and disincentives of following social norms in controversial situations |
| | Balance of Life (PN2.4) (30 min) | To evaluate the social incentives and disincentives of controversial norms; to give an appropriate response to these social issues | To develop the ability to evaluate the incentives and disincentives of following social norms when faced with a controversial issue or situation; to learn to consider the interests of different parties when faced with a controversial issue or situation |
| 9. Resilience | Survivor (RE2.1) (30 min) | To rebuild the understanding of failures and learn from them | To understand that failures may be precursors to success; to find out what one may learn from past failures |

**TABLE 1 (continued)**

| Construct | Unit | Unit Aims | Learning Targets |
|---|---|---|---|
| | The Humorous Moment (RE2.2) (30 min) | To learn to face adversity with a sense of humor so that students can confront difficulties in life optimistically | To learn to face adversity with a sense of humor; to learn the basic principles and skills of humor |
| | The Modern Robinson (RE2.3) (30 min) | To learn not to fall into avoidance traps when facing adversity, as avoidance will only lead to bigger difficulties | To understand why temptations are so tempting and learn skills to resist them; to stay away from short-term happiness due to avoidance |
| | Life Is an Endless Challenge (RE2.4) (30 min) | To learn to take every moment as a learning opportunity and face difficulties and challenges in life courageously | To identify the difficulties and challenges that may help students become mature; to value every moment as a learning opportunity in life |
| 10. Self-determination | Choice: The Panorama (SD2.1) (30 min) | To enhance students' competence in self-determination by sharpening their decision-making skills | To sharpen decision-making skills; to handle peers' opinions and peer pressure by making use of decision-making skills; to understand that one should be responsible for his/her decisions and accept the consequences |
| | Choice: The Prerequisites (SD2.2) (30 min) | To examine each option from different perspectives in order to make a more thorough analysis before deciding | To understand that people may have different perceptions and opinions regarding the same issue; to listen to and analyze the advice of significant others before making a decision |
| 11. Spirituality | Living Elsewhere (SP2.1) (30 min) | To encourage students to live a meaningful life by understanding the living conditions of deprived adolescents | To understand the plight of deprived adolescents; to treasure what we have and live a meaningful life |
| | Money World (SP2.2) (30 min) | To encourage students to reflect on the meaning of money in their lives | To reflect on the meaning of money; to understand that material possessions may not necessarily lead to happiness |
| | To Live a Day (SP2.3) (30 min) | To enable students to reflect on the meaning of life through the experiences of a cancer patient | To become aware that we may encounter challenges in life; to search for the meaning of life through self-reflection |
| | The Beginning and the End (SP2.4) (30 min) | To help students explore the meaning of death through the experiences of a cancer patient | To realize that death is a natural process in life; to reflect on the meaning of death while learning to treasure life |
| 12. Clear and positive identity | Life with Values (ID2.1) (30 min) | To build a positive self-image through discovering the most ideal aspect of oneself | To reveal the ideal "me"; to self-encourage and affirm oneself for the values that one is pursuing |
| | My Companions (ID2.2) (30 min) | To create an atmosphere of mutual support in order to strengthen students' positive self-image | To identify classmates who have similar life values to oneself |
| 13. Beliefs in the future | Go Ahead! (BF2.1) (30 min) | To understand that we have to face our academic results whether they are good or poor; to learn to evaluate ourselves and set short-term learning goals | To learn how to face academic results, as well as how to evaluate ourselves and improve; to set short-term learning goals |

**TABLE 1 (continued)**

| Construct | Unit | Unit Aims | Learning Targets |
|---|---|---|---|
| | Go Forward with Fun (BF2.2) (30 min) | To encourage students to draw up a plan of reward for themselves in order to achieve their learning goals | To understand that rewards are important for achieving goals; to learn how to design an effective plan of reward |
| | Never Give up! (BF2.3) (30 min) | To understand that perseverance is needed for achieving a goal | To understand that perseverance and persistence are needed for achieving a goal; to understand that overcoming temptation brings positive feelings |
| | Make a Wise Change! (BF2.4) (30 min) | To experience and learn how to face and handle obstacles in the process of attaining a goal | To understand that there may be obstacles in the journey of attaining a goal and how to face them with a positive attitude; to learn how to change strategies properly when encountering obstacles and to persist in one's goals |
| 14. Prosocial involvement | The Life Puzzle (PI2.1) (30 min) | To understand the reasons for advocating participation in community activities in society | To familiarize students with programs and services offered by the government and the community for people in need; to understand that people in society have different needs |
| | Hello, Our Community! (PI2.2) (30 min) | To differentiate between the different motives and different factors of participating in community activities | To think about the motives for participating in community activities and the factors affecting such motives |
| | Interactive Community (PI2.3) (30 min) | To learn about the procedures of organizing an activity; to learn to express expectations about participating in community activities | To learn about the procedures of organizing an activity, including identifying aims, setting objectives, and action planning; to express expectations about participating in community activities |
| | School Planting Day (PI2.4) (30 min) | To assess the success of the activities | To evaluate whether an activity achieves its aim; to review the shortcomings of an activity, and to seek ways to improve it in the future |

*Note*: The fifteenth construct of 'Recognition for Positive Behavior' is spread over the other 14 constructs. This contruct is used as teaching strategies and no teaching activity is assigned for this construct. The 10-h Core Program consists of the units from Constructs 1–8. The units RE 2.3 and RE2.4 were observed in School A, the units ID2.1 and ID2.2 were observed in School B, the unit PN2.4 was observed in School C, and the units SD2.1 and SD2.2 were observed in School D. (Schools are identified in Table 2.)

was adopted in the Project P.A.T.H.S. in designing the curriculum units that are intended to cultivate students' development in five different domains: individual, family, peer, school, and society. In the Secondary 2 curriculum, relatively more units are focused on cultivating the students' development in the individual and society domains, e.g., to encourage students to live a meaningful life and to participate in healthy community activities. Moreover, 18 units were designed to tackle some current youth issues, such as (1) mental health problems, such as depressed moods; (2) substance abuse and smoking; (3) heterosexual relationships; and (4) materialism.

There are several lines of evidence that support the effectiveness of the Tier 1 Program of the P.A.T.H.S. Project both in the EIP (EIP-S1: 2005–06; EIP-S2: 2006–07) and FIP (FIP-S1: 2006–07). First, evaluation findings based on the one group pre-/post-test design (EIP-S1: 2005–06; FIP-S1: 2006–07) showed that there were positive changes in the program participants after joining the program[10,11]. Second, subjective outcome evaluation findings based on different studies, sources, and data types showed that the program participants and implementers had positive perceptions of the program and they generally felt that the program was beneficial to the program participants[12,13,14,15,16]. Third, research findings showed a close linkage between subjective and objective outcome evaluation findings, with those perceiving higher benefits of the program showing greater positive changes on the different indicators of positive youth development[17,18]. Fourth, qualitative findings based on focus group interviews showed that the program participants enjoyed the program and they experienced positive changes in themselves[19]. Fifth, interim evaluation findings (EIP-S1: 2005–06 and FIP-S1: 2006–07) showed that the respondents had positive perceptions of the program and its benefits to the program participants[20,21]. Sixth, analyses of the students' weekly diaries showed that the students perceived that the program had helped them in many areas and the participants generally enjoyed the program[22]. Finally, process evaluation studies (EIP-S1: 2005–06 and FIP-S1: 2006–07) based on systematic observations showed that the quality of implementation and program adherence were high[4,5].

Although good implementation quality of the Tier 1 Program was indicated by the process evaluations based on both the EIP (EIP-S1: 2005–06) and FIP (FIP-S1: 2006–07), there was no guarantee that the implementation quality in the EIP on the Secondary 2 Curriculum (EIP-S2: 2006–07) was acceptable. Therefore, process evaluation with systematic observations was carried out to examine the implementation quality of the Tier 1 Program (Secondary 2 Curriculum) based on a random sample of schools for the second year of the EIP (EIP-S2: 2006–07).

## METHODS

### Participants

A total of 49 schools joined the Secondary 2 Curriculum of the Project P.A.T.H.S. in the EIP in 2006/07 academic year (EIP-S2: 2006–07). Among these schools, 21 adopted the 20-h full program that involved 40 teaching units and 28 adopted the 10-h core program that involved 20 teaching units. Among these participating schools, four schools that joined the full program were randomly selected to participate in this study. The characteristics of the schools joining this process evaluation study (Secondary 2 Curriculum) can bee seen in Table 2.

### Procedures

For each school joining the process evaluation study, systematic observations of one or two teaching units were conducted. There were seven units under observation, which covered four positive youth development constructs, including resilience, clear and positive identity, prosocial norms, and self-determination (see Table 2). The learning targets of these units can be seen in Table 1. The observers were two pairs of research assistants of the project who were registered social workers and a colleague with a doctoral degree, with one social worker fixed in each pair. During the observations, each colleague observed how the units were implemented and were required to complete a rating form covering four major areas, including basic information, integration with the school formal curriculum, program fidelity and adherence, and quality of program delivery in an independent manner. For program fidelity and adherence, the observers rated the degree of adherence and recorded the time used to implement the unit. For the quality of program delivery, student interest, student participation and involvement, classroom control, use of interactive delivery method, use of strategies to enhance student motivation, use of positive and supportive feedback, instructors' familiarity with the students, opportunity for reflection, degree of achievement of the objectives, time management, quality of preparation, overall implementation quality, and success of implementation were rated. The research assistants did not have any discussion and they were "blind" to the ratings of their partner when they completed the rating forms. The standardized form for process evaluation can be seen in previous studies[4,5].

**TABLE 2**
**Summary on the Characteristics of Schools Joining the Process Evaluation Study (S2 Curriculum)**

| School | A | B | C | D |
|---|---|---|---|---|
| **Background Characteristics of the Schools:** | | | | |
| Location (district) | Ho Man Tin | Chai Wan | Tai Po | Sham Shui Po |
| Finance mode | Aided | Aided | Aided | Directly subsidized |
| Sex composition | Coeducational | Coeducational | Coeducational | Coeducational |
| Religious background | Nil | Islamism | Christianity | Catholic |
| **Context of Observation:** | | | | |
| Choice of program | 20 h | 20 h | 20 h | 20 h |
| Mode | 20 sessions (1 h/session) | 20 sessions (1 h/session) | 40 sessions (30 min/session) | 20 sessions (1 h/session) |
| Integration with school curriculum | Life education | Life education | Class teacher's period | Life education and leadership |
| No. of students in the class | 38 | 31 | 44 | 24 |
| Instructor(s) | 1 Teacher and 1 social worker | 1 Teacher and 1 social worker | 1 Teacher | 1 Teacher and 1 social worker |
| Teaching medium of the unit observed | Chinese | English | Chinese | English |
| Duration of observation | 70 min | 80 min | 40 min | 80 min |
| Unit(s) observed | RE 2.3 and 2.4 | ID 2.1 and 2.2 | PN 2.4 | SD 2.1 and 2.2 |

## RESULTS

For every unit, the ratings of each item by the two independent observers were averaged. To obtain an overall picture, the ratings for each item across all units were again averaged. In order to test the reliability of the averaged ratings, Spearman correlation was conducted based on the overall adherence ratings across the seven units and the analyses showed that the ratings across the observers in the observed units (N = 7) were highly reliable (rho = 0.97, $p < 0.01$). The average overall adherence to the curriculum manuals was 83.6% (range from 70 to 95%, Table 3), which was quite remarkable. For those units where modifications had been made, the observers generally regarded the changes to be reasonable.

The findings on the program implementation quality can be seen in Table 3. Again, Spearman correlation was conducted based on the mean overall ratings across the seven units and the analyses showed that the ratings across the two observers in the observed units (N = 7) were highly reliable (rho = 0.94, $p < 0.01$). An examination of the different areas of the delivery quality showed that the mean ratings were generally high (over 5 on a 7-point rating scale). In particular, the two observers rated highly positive in the following areas: student participation and involvement, the quality of lesson preparation by the instructors, and the overall implementation quality. The results revealed that the quality of delivery as assessed by the two observers was very good.

## DISCUSSION

The present study limits its scope to one specific aspect of process evaluation, i.e., program monitoring[7,23,24] with a central aim to assess the program fidelity and quality of program implementation. In examining the adherence and quality of implementation of the Tier 1 Program (Secondary 2 Curriculum) of P.A.T.H.S. in the second year of the EIP (EIP-S2: 2006–07), results showed

**TABLE 3**
**Overall Ratings on Each Unit Observed in the Different Schools**

| School | A | | B | | C | D | | Average/Mean |
|---|---|---|---|---|---|---|---|---|
| Unit observed | RE 2.3 | RE 2.4 | ID 2.1 | ID 2.2 | PN 2.4 | SD 2.1 | SD 2.2 | |
| Overall Adherence (%) | 90 | 70 | 80 | 90 | 95 | 90 | 70 | 83.6 |
| **Program Implementation Quality** | | | | | | | | |
| (Ratings on a 7-point scale. The percentages of responses with ratings of 5 and above are in brackets.) | | | | | | | | |
| 1. Student Interest | 6 (100) | 6 (100) | 6 (100) | 6 (100) | 5.5 (100) | 5.5 (100) | 5 (100) | 5.71 |
| 2. Student Participation and Involvement | 6 (100) | 6 (100) | 5.5 (100) | 6 (100) | 6 (100) | 6 (100) | 6 (100) | 5.93 |
| 3. Classroom Control | 4 (0) | 4.5 (50) | 6 (100) | 5.5 (100) | 6 (100) | 5.5 (100) | 5 (100) | 5.21 |
| 4. Interactive Delivery Method | 5 (100) | 5 (100) | 5.5 (100) | 6 (100) | 5.5 (100) | 6 (100) | 6 (100) | 5.57 |
| 5. Strategies to Enhance Student Motivation | 5 (100) | 5 (100) | 5.5 (100) | 6 (100) | 4 (0) | 5 (100) | 5.5 (100) | 5.14 |
| 6. Use of Positive and Supportive Feedbacks | 5 (100) | 5 (100) | 5.5 (100) | 6 (100) | 4.5 (50) | 5.5 (100) | 5.5 (100) | 5.29 |
| 7. Instructors' Familiarity with the Students | 5 (100) | 5 (100) | 6 (100) | 6 (100) | 6.5 (100) | 6 (100) | 6 (100) | 5.79 |
| 8. Opportunity for Reflection | 4.5 (50) | 4.5 (50) | 6 (100) | 5 (100) | 5 (100) | 5.5 (100) | 5 (100) | 5.07 |
| 9. Degree of Achievement of the Objectives | 4.5 (50) | 4.5 (50) | 6 (100) | 6 (100) | 6 (100) | 6 (100) | 6 (100) | 5.57 |
| 10. Time Management | 5 (100) | 5 (100) | 6 (100) | 6 (100) | 6 (100) | 5 (100) | 5 (100) | 5.43 |
| 11. Lesson Preparation | 5 (100) | 5.5 (100) | 7 (100) | 6 (100) | 6 (100) | 6 (100) | 6 (100) | 5.93 |
| 12. Overall Implementation Quality | 5.5 (100) | 5.5 (100) | 6 (100) | 6 (100) | 6 (100) | 6 (100) | 6 (100) | 5.86 |
| 13. Success of Implementation | 5.5 (100) | 5 (100) | 5.5 (100) | 6 (100) | 6 (100) | 6 (100) | 6 (100) | 5.71 |

that the overall degree of adherence to the teaching units assessed by the observers was on the high side. This observation is generally similar to two previous findings[4,5], which showed that the mean program adherence was 84.5% (EIP-S1: 2005–06; range: 50–95%) and 86.3% (FIP-S1: 2006–07; range: 45–100%). The findings of this study suggest a slightly lower program adherence rate of 83.6%, but a much closer range of 70–95% (EIP-S2: 2006–07). In short, the findings suggest that the need for modifying the units in the implementation process was not high and further support that curricula-based positive youth development programs can be easily utilized without major modifications made for different adolescent populations. This conclusion is important because there is a common myth among the program implementers that the program must be substantially modified because it can be meaningfully implemented.

The second major conclusion of the study is that the different aspects of the program delivery were perceived to be very positive. These aspects include: (a) students' interest (item 1) and involvement (item 2, with the highest rating of 5.93); (b) management and teaching strategies used by the instructors (items 3, 4, 5, 6, and 10); (c) instructors' relationship with the students (item 7) and effort in lesson preparation (item 11, also with the highest rating of 5.93). Furthermore, the observers perceived that the objectives of the units implemented could be achieved (item 9; rating: 5.57), the overall quality of implementation was high (item 12; rating: 5.86), and the implementation was successful (item 13; rating: 5.71).

Nevertheless, similar to the previous study[5], the degree of reflection (item 8; rating: 5.07) was the lowest among all items, even though the rating reached 5 in a 7-point scale. The two possible explanations can also be applied in this study, i.e., the overpacking of the curriculum units and the didactic teaching style in Hong Kong. Since reflection is an invaluable part in the learning process that encourages students to evaluate oneself and one's values, which in turn enables their growth and development, it should be further emphasized in subsequent instructors' training.

Among the four schools observed, the scores on curriculum delivery of one school were not good (School A: 4 to 4.5 out of 7). Based on the observations of the observers, several factors may contribute to this situation: less effective classroom control, few opportunities for student reflection in the units, and low degree of achievement of the stated objectives in the two units under observed. A plausible explanation is that the inadequacy in classroom control may have led to the subsequent difficulties in curriculum delivery. This is a factor that should be further explored in future process evaluation studies.

This study had several limitations. First, because of manpower and resource constraints, only four schools were randomly selected to participate in this study and the data collection was carried out by the research team of the Project. It would be desirable to include more schools with different characteristics to participate in the study, and to involve as many staff at every level and stakeholder groups as possible in the day-to-day tasks of program monitoring because it will give everyone a sense of shared ownership in the monitoring process[7]. However, it is noteworthy that data collection must not hinder the functioning of the program and its implementers. Second, only seven curriculum units were involved. Due to limitation of resources, the evaluation design did not provide an ongoing activity to monitor the program to keep it on track, and the evaluators neither collected data over an extended period of time during the course of the program nor conducted ongoing process over the life cycle of the program. Third, besides adherence and the quality of implementation, process evaluation with reference to other dimensions, such as the context of the implementation[25] would help the program developers to further understand the quality of the program implementation process. With reference to the recommendation made by Linnan and Steckler[26] on gaps in current knowledge about process evaluation, future studies should refine on the concept of process, and the related assessment and interpretation methods. Fourth, although the findings revealed that opportunity for reflection among the students was not high, the factors contributing to this observation were not examined. As reflection is an important element in learning and reflective learning has not been emphasized in Chinese students[27], future studies should attempt to examine how Chinese students make sense and conceptualize the role of reflection in their learning in the P.A.T.H.S. Project.

Finally, the generalizability of the present findings should be concerned because the picture of a specific school cannot be shared with other schools because schools are under different management and evaluation results may place a specific school in jeopardy. Also, consistent with the intrinsic problem of all observation studies where time sampling is involved, we need to be conscious of the degree of generalizability of the present findings to other temporal and spatial contexts needs. Of course, this issue was partially addressed by the procedure that the schools were randomly selected from the participating schools. Nevertheless, it is noteworthy that one possible confounding effect is that the students might become more cooperative when there are visitors and outside observers. In addition, it is also possible that the instructors might be more motivated to teach well when being observed. Of course, the use of ethnographic strategies with prolonged engagement and observations would be helpful[28].

Despite these limitations, the existing research findings suggest that the quality of implementation of the Secondary 2 Curriculum of the Tier 1 Program in the EIP was high and the program was helpful to the program participants. Furthermore, the present study acts as an important attempt at program evaluation and also attempts to illuminate certain blind spots that program implementers (school teachers and social workers), who have more personal investment in the program activities, are likely to have. The present findings will be made known to all implementers through publications and future staff training sessions. Similar to all formative evaluations, they are conducted when the program is underway with an aim to improve the overall Project in its FIP as well.

## ACKNOWLEDGMENTS

The preparation for this paper and the Project P.A.T.H.S. were financially supported by the Hong Kong Jockey Club Charities Trust.
